# Factors associated with transplant workup completion in a regional kidney program in Ontario, Canada: a retrospective cohort study

**DOI:** 10.3389/fneph.2026.1789895

**Published:** 2026-05-28

**Authors:** Abdelrahman Elsebaie, Natalie Witton, Wilma Hopman, Arezou Shahmoradi, David Holland, M. Khaled Shamseddin

**Affiliations:** 1 Department of Medicine, Division of Nephrology, Queen’s University, Kingston, ON, Canada; 2 Kingston Health Sciences Centre, Kingston, ON, Canada

**Keywords:** cohort study, dialysis vintage, duration, kidney transplantation, pre-transplant, workup

## Abstract

**Introduction:**

Minimizing delays during the pre-kidney transplant workup is critical for patients and providers. We evaluated the factors associated with workup completion times across the processes and key investigations in our program.

**Methods:**

In this single-center, retrospective cohort study, we included all candidates who initiated their pretransplant workup between January 1, 2019 - December 31, 2022. Patients were allocated, based on their consecutive referrals, alternately to an intensified pretransplant clinic follow-up process with more frequent assessment visits every 4 months during evaluation (Cohort 1), or a single initial assessment with no interim visits until full workup completion (Cohort 2). Follow-up was continued until June 1, 2025. Ethical approval was obtained with waived informed consent.

**Results:**

In total, 207 patients were allocated to Cohort 1 (n = 94, Age 52.2 ± 14.2 years, 45.7% female) and Cohort 2 (n = 113, Age 56.8 ± 12.1 years, 43.4% female), with no significant demographic differences between cohorts. Cohort 1 demonstrated a significantly shorter median workup duration (12.8 vs. 17.1 (-4.1 months), P = 0.007) and reduced dialysis vintage at the time of transplant decision (22.3 vs. 29.4 (-7.1 months), P = 0.056).

**Discussion:**

Frequent pre-transplant assessment visits were associated with expedited workup completion and reduced dialysis vintage.

## 1 Introduction

**Figure 1:**
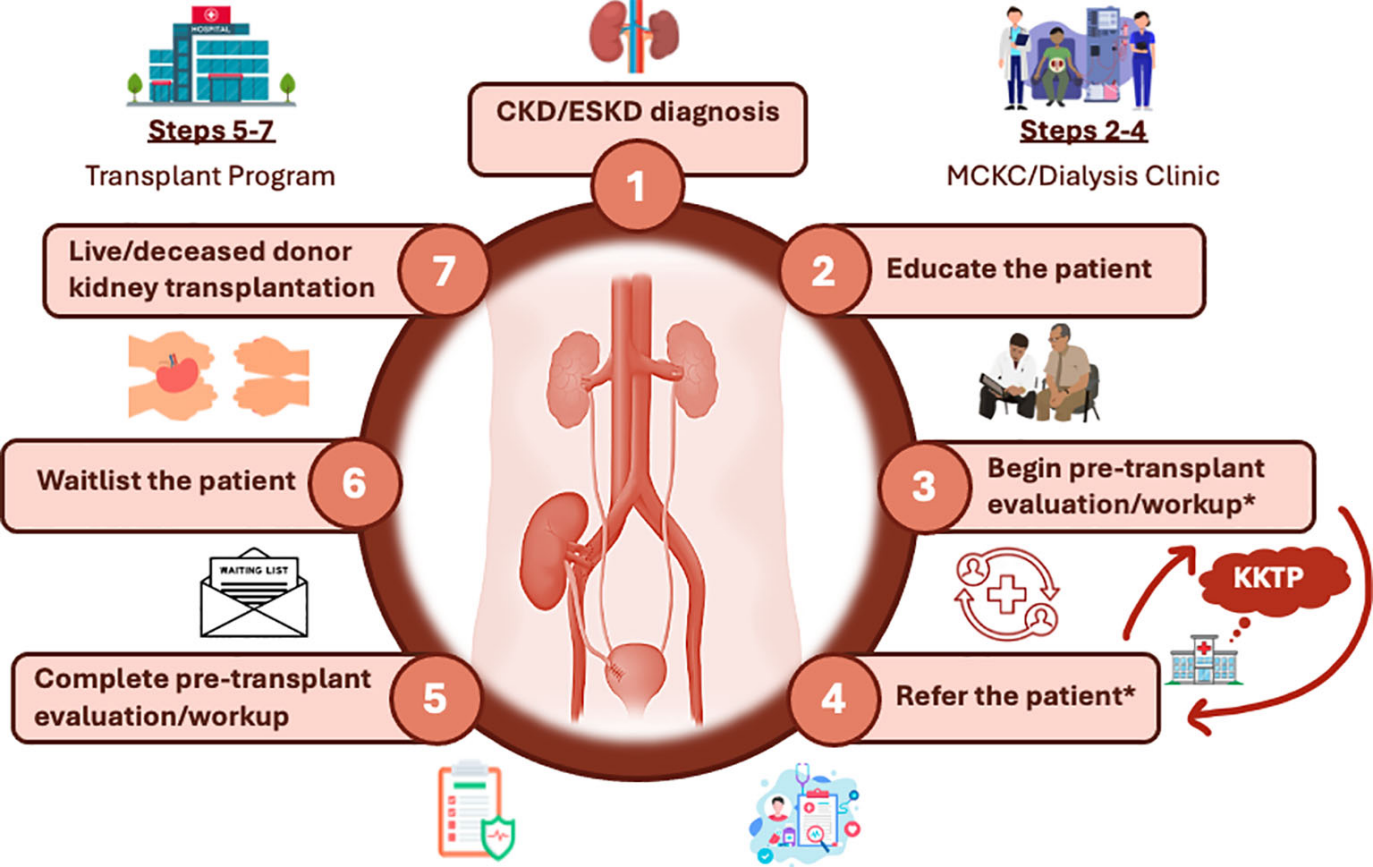
Universal steps of the kidney transplant journey. *For the Kingston Kidney Transplant Program (KKTP), patients’ evaluation/workup begins by the transplant program after being referred (reverse steps 3 & 4). CKD, chronic kidney disease; ESKD, end-stage kidney disease; MCKC, multicare kidney clinic.

Kidney transplantation is strongly linked to enhanced survival (1), improved quality of life (2), and is considered more cost-effective than hemo- or peritoneal dialysis (3). Dialysis vintage prior to transplantation has a proportional adverse effect on patient and graft survival (4–6), and pre-emptive transplantation prior to dialysis initiation is recommended (7, 8). The pre-transplant process begins when the patient, often already on dialysis, opts for the procedure after learning about it as a treatment option (9). This is followed by referral to a transplant center from the primary nephrologist, completion of a comprehensive pre-transplant workup and medical assessment for suitability, and finally concluding with the patient either being placed on a waitlist for a deceased donor or proceeding with live-donor transplantation (9). This process is demonstrated in **Figure 1**.

Failure to move through the transplantation process is usually caused by remaining stationary at specific steps, resulting in a growing cohort of patients not completing their evaluations for listing (10), and a prolonged dialysis vintage contributing to the development of comorbid conditions prior to kidney transplantation (11). The prolonged evaluation process also places significant stress on the limited resources of a transplant center. As new referrals continue to be received, patients in “pending activation limbo” are either neglected or detracted from the evaluation of new candidates (12).

Previous research, including a scoping review of 33 studies, identified several key barriers across multiple levels, including patient- (demographic, socioeconomic, sociocultural, and knowledge), provider- (miscommunication, staff availability, provider perceptions and attitudes), and system- (geography, distance to care, healthcare logistics) level factors (13). Moreover, Indigenous Americans, Black, Pacific Islander, and Hispanic had reduced access to transplant referral and evaluation as compared with White Americans (13). Several studies also reported that distance to a kidney transplant center is a possible barrier to referral and evaluation, likely due to travel time and/or transportation (13).

The Kingston Kidney Transplant Program (KKTP) is one of six adult kidney transplant programs in Ontario, Canada. After referral, the pre-transplant evaluation process is uniquely coordinated by the KKTP rather than by the MultiCare Kidney Clinic (MCKC)/Dialysis programs, which are also part of the same regional renal program in Southeastern Ontario. This process is entirely different from a universal model, in which candidates are usually referred to transplant centers after finalizing their workup to proceed with registration on the waiting list or live transplantation. Although our process could be a cost-effective model for excluding unsuitable patients at earlier stages before a comprehensive, expensive, and time-consuming workup, it could add a tremendous workload to coordinator(s), resulting in a slower workup and a prolonged process, with significant impacts on patients, families, and centers.

Therefore, prior to implementing a different pre-transplant evaluation process in our program, we conducted a retrospective analysis to examine how the frequency of pre-transplant assessment visits influences pre-transplant workup completion times across different processes, investigations, and populations.

## 2 Materials and methods

### 2.1 Study design and participants

This was a single-center, retrospective cohort study of all candidates who initiated their pre-kidney transplant workup between January 1, 2019, and December 31, 2022, at the KKTP located in Southeastern Ontario after implementing an intensified pre-transplant clinic follow-up process in January 2019. Patients were allocated in chronological order of referral using a quasi-randomized assignment approach, whereby consecutive referrals were alternately assigned to either an ‘intensified pre-transplant clinic follow-up process,’ defined as more frequent assessment visits every 4 months until a final transplant decision was reached (Cohort 1) or a ‘standard pre-transplant clinic follow-up process’ (Cohort 2).

In the former process, interim in-person and virtual visits (as requested) primarily served a coordinative function. These encounters allowed the transplant team to update and confirm the patient’s ongoing medical and surgical eligibility for transplantation; review the status of completed and pending investigations; discuss results with patients; identify outstanding components of the workup; arrange additional investigations or specialist referrals as clinically indicated; and address patient questions or logistical barriers to completing the evaluation. On the other hand, the latter process consisted of a single initial pre-transplant assessment, followed by no interim visits until the completion of the full pre-transplant workup, when the completed package was reviewed by the transplant team and a transplant decision was rendered. Meanwhile, the pre-transplant workup was facilitated and reviewed by the transplant coordinator and multidisciplinary team, with additional investigations arranged as needed.

All candidates were followed up until June 1, 2025. The study was conducted in accordance with the Declaration of Helsinki (14), and the Strengthening the Reporting of Observational Studies in Epidemiology reporting guidelines for cohort studies (15). Ethics approval was granted by the Queen’s University Health Sciences and Affiliated Teaching Hospitals Research Ethics Board (protocol code: 6040490; date of approval: March 01, 2024). The requirement for patient consent was waived owing to the retrospective nature of the study.

### 2.2 Data collection and study outcomes

Demographic characteristics were retrospectively extracted from our electronic medical record (EMR) systems (Patient Care System and NephroCare). This included age, sex, race, etiology of chronic kidney disease (CKD)/ESKD, ABO blood group, geographic distance from our center (calculated based on the difference in distance between KGH and patients’ forward sortation area, which is the first three characters of a Canadian postal code), dialysis requirement, and vintage.

Other extracted variables included the date of initial assessment by the transplant nephrologist and the total number of pre-transplant assessment visits. Lastly, we collected the completion dates of time-limiting investigations, namely echocardiography (ECHO), stress test, and colonoscopy, which are usually lengthy, resource-intensive, and necessary assessments of medical suitability.

The primary outcome of interest was the duration of the pre-transplant workup, defined as the time between the transplant assessment date (pre-transplant workup order date in EMR) and the transplant decision date (transplant status update date in EMR). More specifically, the transplant decision date corresponded to the point at which the workup outcome and the patient’s suitability for transplantation were designated into one of eight categories: a) patient placed on the deceased donor list, b) patient proceeded with live donor transplantation, c) patient was medically unsuitable, d) patient needs to be re-assessed at a later time due to relative contraindication(s), e) patient died while in workup, f) patient was no longer interested, g) patient was not committed to the workup and missing appointments (File closed – Nonadherence), and h) patient is still in workup with no transplant decision made to date. The time from dialysis to transplant assessment and decision as well as the duration of ordering to completion of specific investigations (i.e., ECHO, stress test, or colonoscopy) were also computed.

### 2.3 Statistical analysis

The data were imported into IBM SPSS (version 29.0 for Windows, Armonk, New York, 2023) for statistical analysis. Data were initially analyzed descriptively, and the underlying distribution of continuous data was assessed using the Shapiro-Wilk test. The duration of the pre-transplant workup and the duration of individual time-limiting investigations were reported as median (interquartile range), given the non-normal distribution of the data. The patients’ transplant status, dialysis prevalence, vintage, and pre-transplant workup completion in relation to dialysis initiation were reported as counts and percentages. The two groups were then compared using Pearson Chi-square tests (or Fisher’s exact test in the event of cells with fewer than five cases) for categorical data, and the Mann-Whitney U test for continuous data. A p-value of <0.05 was used as the cut-off point for statistical significance, and no adjustment was made for multiple comparisons.

## 3 Results

**Figure 2:**
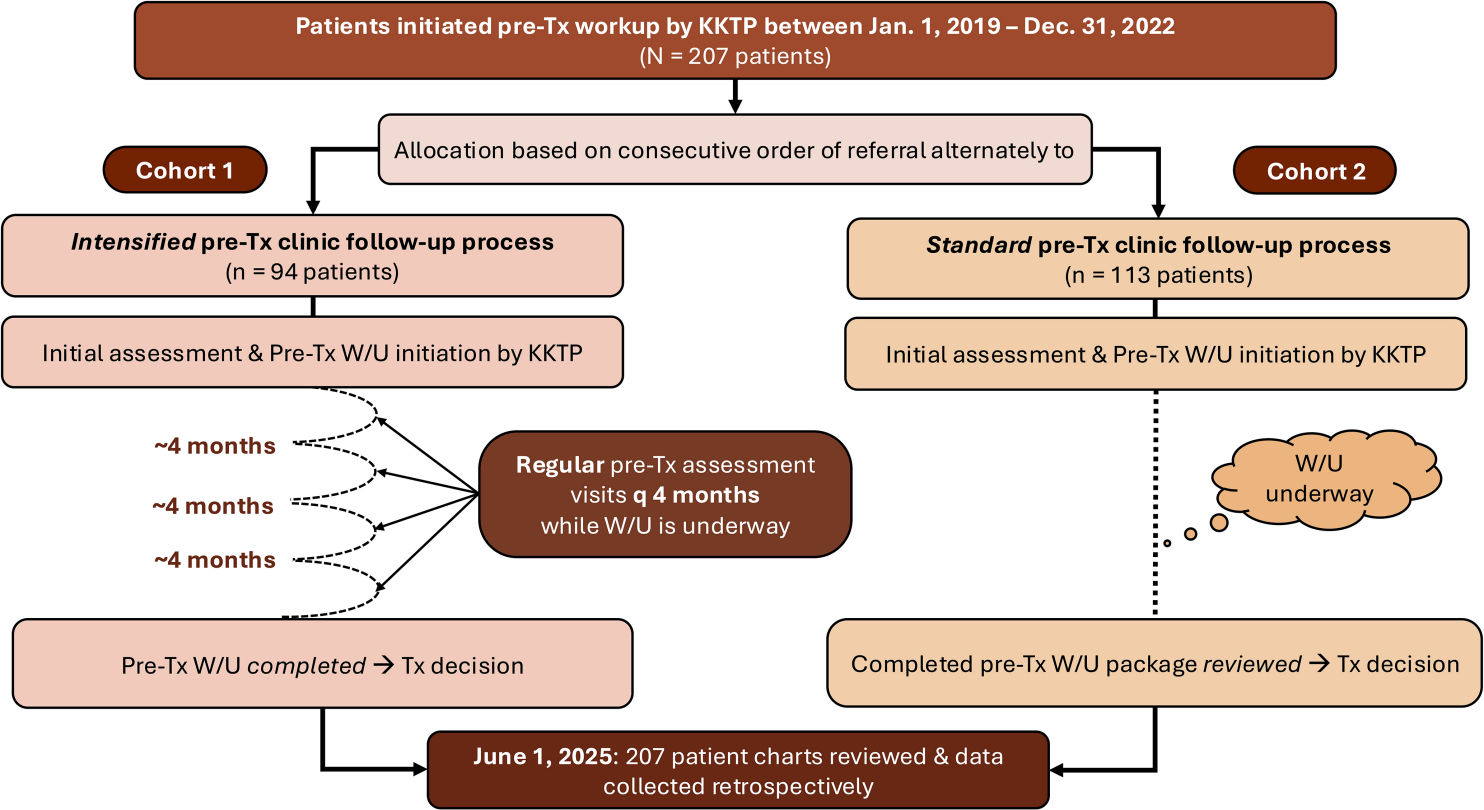
Study flowchart outlining pre-transplant assessment processes. KKTP, Kingston Kidney Transplant Program.

Two hundred and seven patients were evaluated, and their pre-transplant workup was initiated during the study period (January 1, 2019, to December 31, 2022). Based on their chronological referrals (**Figure 2**), 94 patients were allocated to the ‘intensified pre-transplant clinic follow-up process’ (Cohort 1) after excluding 19 patients who never initiated their pre-transplant workup due to personal preference to forgo transplantation or because they no longer met eligibility criteria. The remaining 113 patients were assigned to the ‘standard pre-transplant clinic follow-up process’ (Cohort 2). The patients’ demographic characteristics are summarized in **Table 1**, and no significant baseline differences were observed between the two cohorts. By the time of transplant decision and as expected, a statistical difference was observed in the median number of pre-transplant assessment visits between Cohorts 1 and 2 (3 (3-4) vs. 2 (2-2), *P* < 0.001).

**Table 1:** Patients demographic characteristics.

Baseline Characteristic	Cohort 1 (n = 94)	Cohort 2 (n = 113)	All (N = 207)	*P*
Age, yrs.*, mean (SD)	52.2 (14.2)	56.8 (12.1)	54.7 (13.3)	0.13
Sex, female, n (%)	43 (45.7)	49 (43.4)	92 (44.4)	0.78
Race, n (%)				1.0
Indigenous	15 (16)	17 (15)	32 (15.5)	
All other	79 (84)	96 (85)	175 (84.5)**	
ESKD etiology, n (%)				0.83
Diabetes	38 (40.3)	48 (42.5)	86 (41.5)	
Hypertension	11 (11.7)	11 (9.7)	22 (10.6)	
Glomerulonephritis	27 (28.7)	27 (23.9)	54 (26.1)	
Polycystic kidney disease	4 (4.3)	8 (7.1)	12 (5.8)	
Other	14 (14.9)	19 (16.8)	33 (15.9)	
ABO blood type, n (%)				0.58
A	32 (34)	47 (41.6)	79 (38.2)	
B	14 (14.9)	11 (9.7)	25 (12.1)	
AB	2 (21)	3 (2.7)	5 (2.4)	
O	46 (48.9)	52 (46)	98 (47.3)	
Distance from Tx center, km, median (IQR)	80.4 (7.9-94.8)	80.6 (7.9-96.9)	80.6 (7.9-96.9)	0.65
On dialysis, n (%)				
By June 1, 2025	83 (88.3)	101 (89.4)	184 (88.9)	0.83
Prior to Tx assessment	64 (68.1)	80 (70.8)	144 (69.6)	0.76
Prior to Tx decision	74 (78.7)	91 (80.5)	165 (79.7)	0.26

*Taken at the time of initial Tx assessment.

**Mainly Caucasian (155/175 (88.5%)).

P-values for Cohort 1 vs. Cohort 2 are based on the Pearson Chi-square (or the Fisher’s Exact) for categorical data, and the Mann-Whitney U for continuous data. ESKD, end stage kidney disease; IQR, interquartile range; Tx, transplant.

By June 1, 2025 (**Table 2**), most patients in Cohorts 1 and 2 had received a transplant decision (98.9% vs. 95.6%, *P* = 0.21), while only one patient (1.1%) in Cohort 1 was still in workup compared with five (4.4%) patients in Cohort 2 (*P* = 0.22). Additionally, 78.7% of patients in Cohort 1 required dialysis initiation prior to the transplant decision (**Table 2**) compared to 80.5% in Cohort 2 (*P* = 0.26). That said, a higher proportion of patients in Cohort 1 reached a transplant decision within 1 year and within 1–2 years of dialysis initiation than those in Cohort 2 (18.9% vs. 14.3% and 29.7% vs. 20.9%, respectively). However, these differences were not significant (**Table 2**).

**Table 2:** Transplant decision and patient status.

Transplant Decision/Status	Cohort 1 (n = 94)	Cohort 2 (n = 113)	All (N = 207)	*P*
Tx decision, n (%)				
Still in workup	1 (1.1)	5 (4.4)	6 (2.9)	0.22
Final Tx decision	93 (98.9)	108 (95.6)	201 (97.1)	0.21
Within 1 year of dialysis start	14 (18.9)	13 (14.3)	27 (16.4)	0.31
Within 1–2 years of dialysis	22 (29.7)	19 (20.9)	41 (24.8)	0.31
> 2 years after dialysis start	38 (51.4)	59 (64.8)	97 (58.8)	0.31
Final Tx decision status, n (%)				
Listed	34 (36.2)	39 (34.5)	73 (35.3)	0.88
Proceed with live Tx	14 (14.9)	10 (8.8)	24 (11.6)	0.19
Medically unsuitable	13 (13.8)	17 (15.0)	30 (14.5)	0.85
R/A later	9 (9.6)	8 (7.1)	17 (8.2)	0.61
Death while in workup	4 (4.3)	7 (6.2)	11 (5.3)	0.76
Patient no longer interested	9 (9.6)	11 (9.7)	20 (9.7)	1.0
File closed – *Nonadherence*	10 (10.6)	16 (14.2)	26 (12.6)	0.53

P-values for Cohort 1 vs. Cohort 2; R/A, reassess later; Tx, transplant.

The pre-transplant workup associated with more frequent pre-transplant assessment visits resulted in a lower dialysis prevalence and vintage among Cohort 1 patients at the time of the transplant decision (**Tables 1**–**3**). Specifically, only 51.4% of Cohort 1 patients had been on dialysis for > 2 years, compared with 64.8% of patients in Cohort 2 (*P* = 0.31). At the time of the transplant decision, the distribution of the final transplant decision statuses did not differ significantly between the two cohorts. Nonetheless, Cohort 1 had fewer deaths, file closures, and in-limbo workups (**Table 2**).

**Table 3:** Median (IQR) times of elements of pre-transplant workup.

Time (months)	Cohort 1 (n = 94)	Cohort 2 (n = 113)	Total (N = 207)	*P* (Δ)
Time from Assessment-to-Tx Decision*	12.8 (6.2-20.2)	17.1 (10.1-25.9)	15.6 (8.0-23.8)	0.007 (-4.1 [-24%])
Time from Dialysis-to-Tx Decision	22.3 (11.3-44.3)	29.4 (15.2-56.3)	25.2 (13.4-51.3)	0.056 (-7.1 [-24.1%])
Time from Dialysis-to-Assessment	7.1 (1.4-23.7)	8.6 (3.1-35.3)	8.6 (1.9-28.6)	0.37
Time from Order-to-ECHO	4.1 (1.7-9.1)	4.2 (1.4-7.6)	4.2 (1.5-8.1)	0.76
Time from Order-to-Cardiac Stress Test	6.7 (4.9-10.9)	7.3 (4.6-10.0)	6.9 (4.9-10.2)	0.91
Time from Order-to-Colonoscopy	9.1 (5.2-15.7)	5.5 (2.3-9.5)	6.7 (3.1-13.3)	0.23

*Represents the length of pre-Tx workup.

ECHO, echocardiography; IQR, interquartile range; Tx, transplant; Δ, difference between Cohort 1 & 2 [months (%)].

In those receiving more frequent pre-transplant assessment visits, the median time of pre-transplant workup (time from initial assessment to transplant decision) was significantly shorter in Cohort 1, by 4.1 months, compared with Cohort 2 (12.8 vs. 17.1 months, *P* = 0.007; **Table 3**). The median time from dialysis initiation to transplant decision was also shorter in Cohort 1, by 7.1 months, than in Cohort 2 (22.3 vs. 29.4 months, *P* = 0.056; **Table 3**), even though the time from dialysis initiation to the initial transplant assessment did not differ between cohorts (7.1 (1.4-23.7) vs. 8.6 (3.1-35.3) months, *P* = 0.37; **Table 3**). The median durations of individual time-limiting investigations, specifically the time from ordering to completion of the ECHO, stress test, and colonoscopy, were not significantly different between the two cohorts (**Table 3**).

Finally, there was a clinical signal suggesting that Indigenous patients experienced longer pre-transplant workup durations and greater dialysis vintage at the time of the transplant decision than did non-Indigenous patients (**Table 4**). A similar signal was observed for the median duration of individual time-limiting investigations (ECHO, stress test, and colonoscopy) among Indigenous patients (data not shown). However, the smaller sample size likely contributed to the underpowered data analysis.

**Table 4:** Median (IQR) times of elements of pre-transplant workup – indigenous vs. non-indigenous.

Time (months)	Cohort 1 (n = 94; 15 Indigenous)	Cohort 2 (n = 113; 17 Indigenous)	*P* (Δ)
Pre-Tx Workup			
Indigenous	16.6 (9.1-26.0)	23.3 (11.7-40.1)	0.28 (-6.7)
Non-Indigenous	11.6 (5.9-19.5)	16.7 (9.8-24.3)	0.01 (-5.1)
* P* (Δ)	0.18 (+5.0)	0.96 (+6.6)	
Dialysis-to-Tx Decision			
Indigenous	31.2 (19.5-53.5)	54.2 (21.2-71.1)	0.23 (-23.0)
Non-Indigenous	20.3 (8.9-40.9)	27.4 (14.1-48.3)	0.12 (-7.1)
* P* (Δ)	0.17 (+10.9)	0.03 (+26.8)	
Dialysis-to-Assessment			
Indigenous	12.9 (5.5-33.7)	25.4 (5.1-54.3)	0.57 (-12.5)
Non-Indigenous	6.5 (0.4-20.1)	7.9 (1.6-24.6)	0.42 (-1.4)
* P* (Δ)	0.03 (+6.4)	0.09 (+17.5)	

IQR, interquartile range; Tx, transplant; Δ, difference between cohorts or race (months).

## 4 Discussion

More frequent pre-transplant visits enabled candidates in this ‘intensified pre-transplant clinic follow-up process’ (Cohort 1) to complete their pre-transplant workup within 6.2 to 20.2 months, reflecting an approximate 25% reduction in the workup duration compared with the ‘standard pre-transplant assessment process’ (Cohort 2). This reduction in pre-transplant workup duration was even more pronounced among non-Indigenous patients, with median times of 11.6 vs. 16.7 months, reflecting an approximate 30% decrease.

Our findings are consistent with those of other studies. In a multicenter retrospective study involving a university transplant center in Belgium and 16 referral hospitals where pre-transplant workup was performed, the median time of workup completion was 8.6 (4-14) months (16). In the United States, it takes about 6.7 months to list 50% of evaluated patients and more than 8.2 months to evaluate 75% of patients, with ethnicity and socioeconomic variables as significant contributors (12). In a recently published Canadian single-center quality improvement study, Messina et al. reported a 17% reduction in the median time from transplant evaluation to listing (14.4 (6.8-21.3) vs. 12.1 (7.7-25.9) months) after implementing a multifaceted targeted intervention addressing barriers in pre-transplant workup at their program in Quebec, Canada (17). In comparison, our study showed a significant 25% reduction in pre-transplant workup time, with well-defined workup start and end times (date of transplant workup order and decision date obtained from our EMR system, respectively). Also, it is important to note that the entire pre-transplant workup took place within our transplant program, whereas candidates in the aforementioned study were referred to the transplant program after workup initiation by the CKD/ESKD programs (17). Finally, our ‘intensified pre-transplant clinic follow-up process’ was associated with a shorter median time of stress test completion of 6.7 (4.9-10.9) months compared with 9.1 (2.7-22.3) months in Messina et al.’s study (17). That said, our study did not demonstrate a statistically significant difference in time-limiting investigations between groups. The longer time to colonoscopy completion observed in Cohort 1 is likely attributable to the fact that, although the transplant program can request these investigations, they cannot be directly scheduled.

The coordination challenges identified in our study corroborate system-level barriers previously described in the literature as contributors to delays in pre-transplant workup completion, including geographic distance from the transplant center, limited availability of specialist services in rural and remote areas, poor coordination between referring and transplant programs, and transplant coordinator workload constraints (13). Such barriers are particularly relevant in the context of our program, which serves a geographically diverse catchment area and coordinates the entire pre-transplant evaluation internally. The prolonged dialysis-to-assessment interval that we observed across Cohorts 1 and 2 could be a consequence of these upstream pre-workup system-level obstacles. Thus, our ‘intensified pre-transplant clinic follow-up process’ can be framed as a system-level intervention to mitigate these challenges, irrespective of individual patient circumstances.

Consistent with this, a more frequent pre-transplant assessment process was associated with a shorter dialysis duration among Cohort 1 patients. At the time of the transplant decision, only half of Cohort 1 patients required dialysis beyond two years, compared with 65% in Cohort 2. The median time from dialysis to transplant decision in Cohort 1 patients was also shorter than that in the Cohort 2 candidates. Dialysis vintage can be further reduced by earlier referral to transplant, given that the upper quartile (Q3) time from the start of dialysis to the initial assessment reached 23.7 and 35.3 months in Cohorts 1 and 2, respectively (**Table 3**).

When examining subgroups, Indigenous patients were disadvantaged during their pre-transplant workup compared with non-Indigenous candidates, irrespective of the assessment process (**Table 4**), resulting in substantially longer 1) pre-transplant evaluation times (5 months longer in Cohort 1 and 6.6 months in Cohort 2); 2) dialysis vintage at the time of transplant decision (10.9 months longer in Cohort 1 and 26.8 months in Cohort 2); and 3) time from dialysis to the initial transplant assessment (6.4 months longer in Cohort 1 and 17.5 months in Cohort 2). That said, the ‘intensified pre-transplant clinic follow-up process’ was associated with substantially shorter durations of pre-transplant workup (-6.7 months) and reduced dialysis vintage (-23 months) at the time of the transplant decision among Indigenous patients compared with cohort 2 (**Table 4**).

Particularly, the ‘standard pre-transplant clinic follow-up process’ (Cohort 2), which involved less frequent assessment visits, was associated with a significantly prolonged dialysis vintage (+26.8 months) at the time of the transplant decision among Indigenous patients compared with non-Indigenous patients (**Table 4**). This disparity is likely attributable to the greater need for enhanced communication, greater distance from the transplant program, and delays in rescheduling missing investigations. Similarly, the median times to complete the ECHO, stress test, and colonoscopy were longer in Indigenous versus non-Indigenous patients, with shorter completion times associated with the ‘intensified pre-transplant clinic process,’ although these differences were not statistically significant (data not shown).

To evaluate the impact of the COVID-19 pandemic on pre-transplant workup time, given the reallocation of scarce hospital resources, we conducted a *post-hoc* analysis limited to patients who initiated their pre-transplant workup in 2019 and 2022. This analysis did not identify any significant effects of the pandemic on any of the measured outcomes (data not shown).

Our study has several limitations, including its retrospective design, small sample size, potential COVID-19 pandemic-related delays in the pre-transplant workup, and possible selection bias due to the quasi-randomized allocation of patients to either cohort/process by the recipient coordinator. Nonetheless, the two cohorts were highly comparable with respect to key demographic characteristics. Likewise, the allocation process, although not formally randomized, assigned patients alternately to either cohort/process based on their consecutive order of referral. Also, a formal analysis of patient- and program-level costs, as well as the duration of each step in the pre-transplant workup process, was beyond the scope of this study. Future *post hoc* analyses may help identify rate-limiting steps beyond the time-limiting investigations examined here, specifically those managed outside the transplant program, where the program can request expedited processing but cannot directly control scheduling.

Finally, while our findings suggest that more frequent pre-transplant assessment visits are associated with faster workup completion and reduced dialysis vintage, this approach may be difficult to implement in large-volume centers due to capacity constraints, competing demands, and delays in initial evaluation. In some jurisdictions, long wait times for the first transplant visit may further limit the impact of intensified follow-up. As such, our findings may be most applicable to centers with sufficient infrastructure and staffing to support more frequent patient engagement. Additionally, these limitations highlight the importance of optimizing the pre-referral phase. For example, early initiation of basic workup by referring nephrologists and improved communication with transplant centers can help reduce delays and resource burden. Standardized referral pathways, digital health tools, and shared-care or decentralized models may offer practical, scalable strategies to improve efficiency and timeliness of kidney transplant evaluation, particularly in high-volume settings.

Further research focused on patient-centered, cost-effective models is also needed in this area. Artificial intelligence (AI) chatbots and AI-driven models or applications can play a crucial role in streamlining pre-transplant workups. These tools could send automated reminders for critical investigations and appointments to both patients and coordinators, rescheduling missed tests and visits and improving overall logistics. Adopting such technologies in transplant programs may help expedite pre-kidney transplant evaluations, enhance patient communication with medical teams, reduce delays for candidates, shorten pre-transplant workup duration and dialysis vintage, and improve clinical outcomes.

In conclusion, our study demonstrated that implementing an ‘intensified pre-transplant clinic follow-up process,’ operationalized as more frequent pre-transplant assessment visits, was associated with significantly shorter pre-transplant workup duration and reduced dialysis vintage.

## Data Availability

The raw data supporting the conclusions of this article will be made available by the authors, without undue reservation.
